# Enhanced Stability of Lipid Structures by Dip-Pen Nanolithography on Block-Type MPC Copolymer

**DOI:** 10.3390/molecules25122768

**Published:** 2020-06-15

**Authors:** Hui-Yu Liu, Ravi Kumar, Madoka Takai, Michael Hirtz

**Affiliations:** 1 Institute of Nanotechnology (INT) & Karlsruhe Nano Micro Facility (KNMF), Karlsruhe Institute of Technology (KIT), Hermann-von-Helmholtz-Platz 1, 76344 Eggenstein-Leopoldshafen, Germany; hui-yu.liu@kit.edu (H.-Y.L.); ravi.kumar@kit.edu (R.K.); 2 Department of Bioengineering, The University of Tokyo, 7-3-1 Hongo, Bunkyo-Ku, Tokyo 113-8656, Japan; takai@bis.t.u-tokyo.ac.jp

**Keywords:** MPC copolymer, lipid dip-pen nanolithography, microchannel cantilever spotting, phospholipids

## Abstract

Biomimetic lipid membranes on solid supports have been used in a plethora of applications, including as biosensors, in research on membrane proteins or as interfaces in cell experiments. For many of these applications, structured lipid membranes, e.g., in the form of arrays with features of different functionality, are highly desired. The stability of these features on a given substrate during storage and in incubation steps is key, while at the same time the substrate ideally should also exhibit antifouling properties. Here, we describe the highly beneficial properties of a 2-methacryloyloxyethyl phosphorylcholine (MPC) copolymer for the stability of supported lipid membrane structures generated by dip-pen nanolithography with phospholipids (L-DPN). The MPC copolymer substrates allow for more stable and higher membrane stack structures in comparison to other hydrophilic substrates, like glass or silicon oxide surfaces. The structures remain highly stable under immersion in liquid and subsequent incubation and washing steps. This allows multiplexed functionalization of lipid arrays with antibodies via microchannel cantilever spotting (µCS), without the need of orthogonal binding tags for each antibody type. The combined properties of the MPC copolymer substrate demonstrate a great potential for lipid-based biomedical sensing and diagnostic platforms.

## 1. Introduction

Phospholipid membranes play a key role in living systems, as they are the way cells delineate themselves from the outside built compartments for different functions within the cell and are even used for communication purposes [1,2]. This eminent role leads to big interest in artificial biomimetic phospholipid membranes that have then been used in biosensors [3,4,5], membrane-protein research [6] and other biological applications [7,8]. Supported lipid bilayers (SLBs) are a subset of these biomimetic membranes that is particularly interesting for arraying and studies of biological interactions [9]. While there are a variety of different methods to generate SLBs, arbitrarily shaped and highly localized structures are still challenging to produce [10]. Here, dip-pen nanolithography (DPN) [11,12] with phospholipids (L-DPN) [13] emerged as a novel approach for the rapid fabrication of large-scale phospholipid nanostructure on a variety of substrates [14]. In L-DPN, the DPN setup precisely controls the tip of an atomic force microscope (AFM) cantilever (or an array of such cantilevers) coated with a phospholipids inks to deposit the lipid mixture in desired shapes onto desired substrate locations. Controlling process parameters, like the ambient humidity, scan speed of the AFM tip and contact force allows to obtain the desired thickness of supported lipid membranes by depositing single to many-layer membranes [13,15].

In many applications, the stability of the lipid layers in liquid is an issue, considering the multiple incubation and washing steps in buffers in a typical biomedical experiment. As phospholipids are of amphiphilic nature, and the phospholipid membranes are only physisorbed on the substrates, the substrate properties, such as hydrophilicity, hydrophobicity, surface energy and roughness of surface, can all influence the diffusion, spreading and organization of supporting lipid membranes [16,17,18,19,20]. While tethering membranes can be one way to improve stability, it can also complicate the setup and introduce additional changes to a SLB, in comparison to biological membranes [21,22]. Therefore, other ways of improving the stability of L-DPN-generated structures by tuning the substrate properties are of interest.

Recently, a random copolymer of 2-methacryloyloxyethyl phosphorylcholine (MPC), 3-methacryloxypropyl trimethoxysilane (MPTMSi) and 3-(methacryloyloxy) propyl-tris(trimethylsilyloxy) silane (MPTSSi) was synthesized for improved hemocompatibility of polydimethylsiloxane (PDMS) substrates [23]. PDMS-based polymers are widely used in medical devices; however, the hydrophobicity of PDMS causes unfavorable reactions, such as blood-clotting, protein adsorption and so on. The novel MPC copolymer improved the hemocompatibility after being covalently linked to the PDMS surface by silane coupling (crosslinking) and hydrophobic interactions (mediated by the MPTMSi and MPTSSi moieties, respectively), significantly reducing protein adsorption [23]. Interestingly, the MPC unit in the copolymer that is responsible for the enhanced hemocompatibility is highly analogous in structure to phospholipid headgroups. This prompts us to trial the MPC copolymer with crosslinking as promising substrates for L-DPN generated lipid membrane structures, especially in the context of biomedical experiments involving many washing steps.

## 2. Results

### 2.1. Characterization of Polymer Substrates

**Figure 1:**
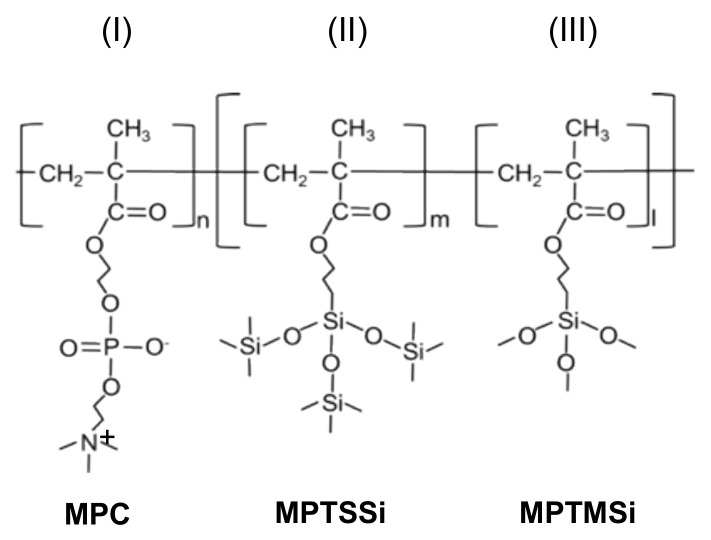
Structure of the copolymer used as substrate. The three functional moieties are (**I**) a phospholipid-like unit 2-methacryloyloxyethyl phosphorylcholine (MPC), (**II**) a hydrophobic unit 3-(methacryloyloxy) propyl-tris(trimethylsilyloxy) silane (MPTSSi) and (**III**) a substrate bonding unit 3-methacryloxypropyl trimethoxysilane (MPTMSi).

The chemical structure of the improved MPC copolymer is illustrated in Figure 1. The block-type MPC copolymer improves the water wettability, as compared with the previously reported MPC copolymer with crosslinking [23]. The unit ratio of MPC:MPTSSi:MPTMSi in the block-type MPC copolymer was 158:18:16, and the molecular weight was 8.4 × 10^4^, respectively. To generate the MPC surfaces, first substrates (SiO_2_ and glass depending on experiment) were washed by ultrasonic cleaning in hexane, ethanol and acetone, before treatment by oxygen plasma. The polymer solution (0.2 wt.% in ethanol) was mixed with 0.1 M acetic acid (10 wt.%). Then, the substrates were immersed in the MPC copolymer solutions for 2 h, dried in solvent vapor atmosphere, at room temperature, for 2 h, and finally heated to 70 °C for 3 h.

As a first step, the different substrate types used in this study were characterized for roughness and contact angle. Here we compare plasma-cleaned standard glass cover slips (plasma-cleaned four days prior to use in experiment to obtain mildly hydrophilic surfaces), silicon substrates and silicon substrates covered with the block-type MPC copolymer. The silicon substrates were commercially available substrates for surface-enhanced ellipsometric contrast (SEEC) microscopy with a silicon oxide top layer, used without further surface treatment [24]. They were previously utilized in L-DPN studies [25] and therefore chosen as further comparison surface. Roughness as obtained by AFM measurement and the static contact angles with water are shown in Table 1 and Figures S1 and S2 for all three surfaces.

The results show that the block-type MPC copolymer has an intermediate roughness of (0.43 ± 0.07) nm, compared to the smoother silicon oxide surface with (0.22 ± 0.02) nm and the rougher glass surface with (1.05 ± 0.08) nm, respectively. The hydrophilicity, as indicated from the water contact angle (WCA), shows the block-type MPC copolymer to be the most hydrophilic substrate with a WCA of (25.5 ± 1.8)°, followed by the aged plasma-cleaned glass with (27.5 ± 1.2)° and the untreated silicon oxide surface with (61.9 ± 3.3)°.

### 2.2. Comparison of Lipid Writing on the Different Substrates

**Figure 2:**
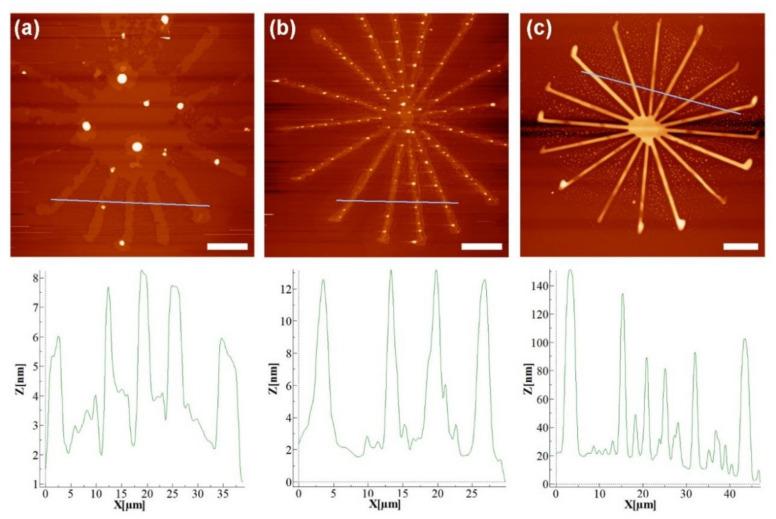
Typical outcomes of L-DPN on the different substrates. AFM images (topography, tapping mode) of star-shaped lipid structures on (**a**) glass, (**b**) SiO_x_/SEEC substrate and (**c**) block-type MPC copolymer. The profile lines in the bottom row show the respective height profile corresponding to the blue line in the AFM images. Scale bar equals 10 µm in all images.

To compare the stability of lipid structures on the different substrates, star-shaped test patterns were written onto each substrate type by L-DPN and subsequently inspected with AFM. For this, a mixture of DOPC with 5 mol% of biotin-Cap-PE and 1 mol% Rho-PE was used. The stars consist of eight crossed lines with a length of 60 µm each. Representative outcomes of the L-DPN writing are shown in Figure 2.

The most pronounced spreading was observed on the glass substrate, and on both, the plasma-treated glass and the SiO_x_/SEEC surfaces only comparably thin lipid membrane stacks (8–12 nm corresponding to around 2–4 lipid bilayers) were observed. In contrast, much higher stable structures resulted on the block-type MPC copolymer: Here, lipid membrane stack heights reached up to 140 nm (around 46 lipid bilayers); see a direct comparison of height in Figure S3. On visual inspection, the star patterns are more homogeneous and clearer on the block-type MPC copolymer, compared to the other substrates. Smaller lipid droplets are observed in between the lines of the structure on the MPC copolymer. These are probably originating from lipid material displaced from the (in comparison to the other substrates higher) already-written lines, when the next line is added to the star shape. This material is then randomly coalescing into droplets on the area between the lines. The average line width for the block-type MPC copolymer on SiO_2_ substrates was (2.5 ± 0.1) µm, while the other substrates showed broader lines of (5.5 ± 0.2) µm (SiO_x_/SEEC) and (6.8 ± 0.7) µm (glass), respectively (Table 2).

### 2.3. Stability of Lipid Arrays in Liquid

**Figure 3:**
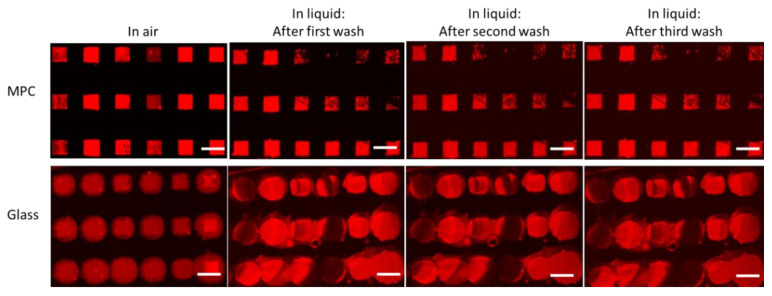
Stability of lipid arrays on block-type MPC copolymer and glass samples. The images show fluorescently labeled lipid patch arrays in air and after three subsequent washing steps on block-type MPC copolymer (top row) and glass (bottom row). The arrays on glass show pronounced spreading on the first washing step. All scale bars equal 50 µm.

As most experiments related to biomedical research take place in liquid and involve several washing steps, arrays of lipid patches were trialed for stability upon washing on block-type MPC copolymer and glass surfaces. Additionally, transparent substrates are favored in most biomedical experiments, as they allow for observation of experiments via inverted microscopes through the substrate. Hence, in the following, we compare the transparent substrates of glass and MPC-copolymer-covered glass. Figure 3 shows the results of three subsequent washing steps. Each washing step consists of 10 times of dipping the sample into DI water.

Here, arrays of 30 × 30 µm^2^ square-shaped patches of DOPC with 1 mol% Rho-PE were prepared by L-DPN, on block-type MPC copolymer substrates and glass. As expected from the previous results, the structure on glass show some spreading, leading to a more roundish shape instead of the clear square structures that result on the block-type MPC copolymer. Upon the first washing step, the structures substantially spread out on the glass substrates, to a point where the single array features start to merge. It should be noted that spreading and reorganization take place particularly on the first washing. Subsequent washing steps did not significantly change the array features any more, indicating that the features spread out to the extent of a single bilayer membrane and formed a SLB. In contrast, the arrays on the block-type MPC copolymer remained undisturbed on washing, and neither on the first nor later washing steps was spreading observed, and clear square shapes were maintained. Instead, the fluorescence intensity of some array features reduced, indicating that, here, instead of spreading, the first washing step could introduce the loss of upper layers in the membrane stack. On subsequent washing steps, the features remained stable in shape and intensity (Figure S4).

### 2.4. Multiplexed Immobilization of Antibodies

**Figure 4:**
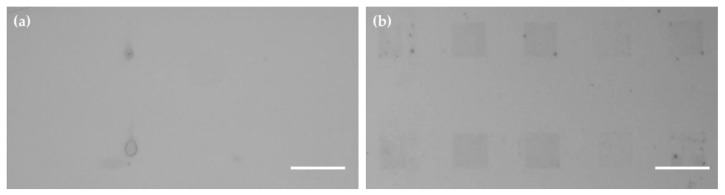
Visibility of lipid patch arrays in bright field microscopy. Array of 30 × 30 µm^2^ square-shaped lipid patches (**a**) on glass and (**b**) on block-type MPC copolymer substrates. While the lipid membrane stacks are visible on the block-type MPC copolymer due to their relative higher thickness, the array features are not discernable on the glass substrate. Here, only thick droplets of lipid are visible. Scale bars equal 50 µm.

The high stability of lipid arrays on block-type MPC copolymer substrates also allows for in situ modifications of the phospholipid membranes. First of all, the thicker membrane stacks on the block-type MPC copolymer become visible in normal bright field microscopy, therefore making it much easier to target them for subsequent modifications (Figure 4).

**Figure 5:**
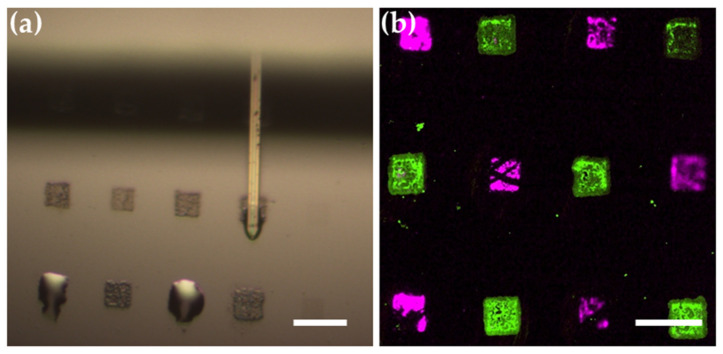
Multiplexing immobilization of antibodies on a lipid membrane patch array. (**a**) The optical microscopy image shows good stability and in situ visibility of the lipid membrane patches on the block-type MPC copolymer surface allows for easy addressing and directly spotting of antibodies with the µCS probe. Two membrane stacks in the lower visible array row are already covered with the incubation droplet. (**b**) The fluorescence microscopy image shows subsequent immuno-staining with secondary antibodies and reveals the successful multiplexed immobilization of EpCAM antibody (green) and Annexin A1 antibody (purple). Scale bars equal 20 µm.

To demonstrate the added functionalization possibilities enabled by the stability and easier visibility of lipid membrane stacks on the block-type MPC copolymer, we trialed the multiplexed immobilization of two different antibodies via microchannel cantilever spotting (µCS). In µCS, a cantilever carrying a microchannel connected to an on-chip ink reservoir is brought into contact with a substrate which then allows transfer of liquid via capillary forces [28,29]. Here, µCS was used to spot two different biotinylated antibodies (Annexin A1 antibody from rabbit and EpCAM antibody from mouse) directly on lipid membrane patches. The lipid membrane patches were written via L-DPN in 10 × 10 µm^2^ squares, with an ink containing biotinylated lipids (DOPC with 5 mol% biotin-Cap-PE). The lipid patch arrays were first homogeneously incubated with streptavidin from solution, to provide binding sides to the biotinylated antibodies via a biotin–streptavidin–biotin sandwich structure [30,31]. Then, on the dried sample, the individual patches were incubated with the respective biotinylated antibody via µCS (Figure 5a).

After the µCS procedure, the sample is washed again, to remove excess of antibody, and the successful immobilization of the antibodies is probed via immuno-staining with secondary antibodies. For this, fluorescently labeled antibodies (anti-mouse IgG labeled with Alexa Fluor 488 targeting the mouse-derived EpCAM antibody and anti-rabbit IgG labeled with Alexa Fluor 647 targeting the rabbit-derived Annexin A1 antibody) are incubated, and the resulting fluorescent pattern is observed (Figure 5b). The resulting distinct fluorescence on the array shows that the immobilization can target specific membrane stack without cross-contamination and that the array is stable under the subsequent incubation and washing steps.

## 3. Discussion

The combined results show that the block-type MPC copolymer is an attractive substrate for L-DPN applications, particularly when thicker membrane stacks are desired. The star-shaped test pattern showed that the block-type MPC copolymer substrate can offer comparatively high lipid structures in good fidelity, without excessive spreading, despite its high hydrophilicity. Usually, stable structures of higher lipid membrane stacks require hydrophobic substrates [20,32]; however, for many biomedical applications, a hydrophilic substrate is often preferred for reasons of biocompatibility or anti-fouling/protein resistance. On hydrophilic substrates, strict control in membrane stack height is needed, to ensure that the transformation into a SLB upon transfer into liquid preserves pattern fidelity and avoids deterioration of patterns by excessive spreading of material from upper layers in the membrane stack [33]. The use of thin membrane stacks and SLBs is fine for the presentation of proteins or similar cell-interface applications [34,35,36]; applications where the lipid patches act as specific binding points on surfaces, e.g., for protein crystallization [37]; or when the lipid acts as carrier matrix for other small molecules that should be delivered to the surface [38,39].

Additionally, SLBs have been widely applied in biomedical research, such as biosensors [4] and membrane protein research [6]. The MPC copolymer can maintain the shape of lipid features in air and in liquid better; thus, interference of lipid spreading with the desired functions in such experiments can be avoided. Additionally, thicker membrane structures are desired, in particular, in sensing applications, as there, thicker structures can boost signal strength and, hence, sensitivity, by providing more sensor material [32,40]. Here, the block-type MPC copolymer offers, by the virtue of the phospholipid-headgroup-like MPC block, a hydrophilic and even hemocompatible alternative substrate still hindering spreading in higher structures. Moreover, the thicker lipid stacks allow for better visibility of these structures in optical microscopy, enabling downstream functionalization with antibodies or easier targeting of the stacks, with possible use as in capture applications, like, for example, Wu and colleagues, who immobilized anticancer antibodies on a SLB, to capture circulating tumor cells (CTCs) [41]. On the MPC copolymer, we demonstrated that the lipid stacks can be directly targeted for immobilization of antibodies in a multiplexed fashion, which can improve the efficiency and versatility of such biosensors.

The exact reason for the higher stability on the MPC copolymer remains a question for future research. Generally, lipid spreading on surfaces is mostly affected by hydrophilicity and roughness [42,43,44,45,46]. In the MPC copolymer case, the enhanced stability of the lipid structures is probably caused by the zwitterionic phosphorylcholine group in the polymer side chain. This enables a unique interaction of the polymer with the matching similar headgroups in the lipid membrane in comparison to other substrates [23,43,46,47]. While the MPC copolymer here is expected to be slightly less densely packed than polymer brushes prepared by surface-initiated atom-transfer radical polymerization (SI-ATRP), it will probably be similarly hydrated and swollen [48] and therefore expected to be even smoother in liquid than in air. Finally, charge effects can play a role in the interaction, as the lipid mixtures used in our study carry negative partial charges. The block-type MPC copolymer itself is a very stable coating for the substrate in regard to washing, as it is crosslinked covalently to the SiO_2_/glass substrates. For the lipid structures on top, the washing experiments presented here show that the higher lipid structures on the block-type MPC copolymer are even stable under subsequent liquid exchange, hence being in particular suited for biomedical detection procedures that usually involve several washing steps. Finally, the enhanced stability and visibility of the membrane stacks on the block-type MPC copolymer facilitates multiplexed functionalization of lipid membranes with antibodies, without the need of specific binding sites for each antibody type. This is achieved by selective delivery of antibodies via µCS on the desired array positions. Instead of needing a specific binding motive per type of antibody (e.g., polyhistidine- and biotin-tags [34]) with the implicit limitation in the number of available orthogonal tags, by this direct targeting, all antibodies can carry the same tag, e.g., the commercially widespread biotin-tag. Thus, the stability of the lipid membrane stacks and being able to reposition spotting tools precisely over the lipid patches enables functionalization not possible by homogeneous self-assembly from solution.

## 4. Materials and Methods

### 4.1. Materials

Phospholipids used in the study were 20 mg mL^−1^ 1,2-dioleoyl-sn-glycero-3-phosphocholine (DOPC), 25 mg mL^−1^ 1-oleoyl-2-(12-biotinyl(aminododecanoyl))-sn-glycero-3-phosphoethanolamine (18:1 -12:0 Biotin-CAP-PE) and 1 mg mL^−1^ 1,2-dioleoyl-sn-glycero-3-phosphoethanolamine-N-(lissamine rhodamine B sulfonyl) (18:1 Liss Rhod PE) all solved in chloroform (Avanti Polar Lipids, Alabaster, AL, USA). Antibodies used were rabbit polyclonal biotinylated Annexin A1 antibody, mouse monoclonal biotinylated EpCAM antibody, goat anti-mouse IgG H&L (Alexa Fluor 488) and donkey anti-rabbit IgG H&L (Alexa Fluor 647) (Abcam, UK). Streptavidin was purchased from Sigma-Aldrich, Taufkirchen, Germany. MPC was purchased from NOF Co. (Tokyo, Japan), and 3-(Methacryloyloxy) propyl-tris(tri(methylsilyloxy) silane (MPTSSi) and 3-methacryloxypropyl trimethoxysilane (MPTMSi) were purchased from Shin-Etsu Chemical Co. (Tokyo, Japan). Ethanol, hexane, 1-propanol, acetone, diethyl ether, acetic acid and 1,1,1,3,3,3-hexafluoroisopropanol (HFIP) were purchased from Wako Pure Chemical Industries, Ltd. (Osaka, Japan). These chemicals were used without purification. Moreover, α,α′-Azobisisobut yronitrile (AIBN), ethanol-d_6_ for NMR and deuterium oxide-d_2_ for NMR were purchased from Sigma–Aldrich (St. Louis, MO, USA). We purchased 4-Cyano-4-(phenylcarbonothioylthio)pentanoic acid (CPD) from Strem Chemicals, Inc. (Newburyport, MA, USA).

### 4.2. Preparation of MPC Copolymer Substrates

Poly(MPC-*b*-(MPTSSi-*r*-MPTMSi)) denoted as block-type MPC copolymer (Figure 1) was synthesized by the two-phase RAFT polymerization process. The MPC monomer (0.5 M), AIBN as initiator and CPD as RAFT agent were dissolved in 1-propanol, and polymerization was performed at 65 °C for 24 h. Without reprecipitation, the second phase of copolymerization was performed. MPTSSi, MPTMSi and 1-propanol were added to the poly(MPC). Polymerization was performed at 65 °C for 24 h, and the block copolymers were then collected by reprecipitation in diethyl ether and hexane (7/3, *v*/*v*). The block-type MPC copolymer was not dried but dissolved in ethanol.

The chemical structure of the block-type MPC copolymers was identified by proton nuclear magnetic resonance (^1^H-NMR; JNM-GX270, JOEL, Tokyo, Japan). D_2_O was used for poly(MPC), and Ethanol-d_6_ was used for the others. The weight-averaged molecular weights (M_w_) of the block-typed MPC copolymer were determined by gel permeation chromatography (GPC; JASCO RI-1530 detector, Tosoh Co, Tokyo, Japan, Column; TSK-GEL Super HM-M, solvent: HFIP, flow rate: 0.2 mL/min). Poly (methyl methacrylate) was used as a standard.

### 4.3. Contact Angle Measurement

The static contact angles of water droplets on the MPC surfaces were recorded at room temperature, using an OCA-20 contact angle analyzer (DataPhysics Instruments GmbH, Filderstadt, Germany). Five water droplets with same volume and rate (2 µL and 2 µL s^−1^, respectively) were dropped on the surface, in different areas, and the average value of the measured contact angle was reported.

### 4.4. Atomic Force Microscopy

AFM was done on a Dimension Icon instrument from Bruker (Karlsruhe, Germany). AFM tips with a spring constant of 40 N m^−1^ and a nominal resonance frequency of 325 kHz were used (type HQ:NSC15/Al BS from MikroMasch, Wetzlar, Germany). Tapping mode was used for topographic images of the star-shaped structures, at a scan rate of 0.5 Hz and 512 samples per line. Sample roughness was obtained from 5 × 5 µm^2^ scans, with a scan rate of 0.7 Hz and 768 samples per line in tapping mode. The quantification of the AFM data was done on WSxM 4.0 beta 9.1 [49] and Nanoscope analysis v1.4 software (Bruker, Karlsruhe, Germany). The roughness value was obtained after flattening the image as R_q_ value in the roughness section of the Nanoscope analysis software. For height profiles, derived data were obtained of the image in the WSxM software, after flattening the image and drawing the corresponding profile line across the pattern. The height profiles in Figure 2 were smoothened in WSxM, after evaluation for better graphical quality.

### 4.5. Writing of Lipid Structures and Functionalization

#### 4.5.1. Writing of Lipid Structures by L-DPN

The lipid pattern arrays were written by L-DPN on a DPN 5000 system (NanoInk, Skokie, IL, USA) with M-type cantilevers (ACST, Carlsbad, CA, USA) featuring 12 parallel tips in a 66 µm pitch. The tips were homogenously coated with the desired phospholipid mixture (DOPC with admixing of 1 mol% Rhod PE and/or 5 mol% Biotin-CAP-PE) by dipping into matching inkwells (ACST, Carlsbad, CA, USA) for 5 min, in a controlled environment of 60% relative humidity (RH). Afterward, the desired lipid patterns were written onto the respective substrates in 35–45% RH and with a writing speed of 0.2 to 2 μm s^−1^. All L-DPN experiments were performed at room temperature.

#### 4.5.2. Multiplexed Immobilization of Antibodies

First, the phospholipid arrays containing DOPC with 5 mol% Biotin-Cap-PE were incubated for 30 min, with PBS, to which 0.5 vol% streptavidin (stock solution of 1 mg mL^−1^ in PBS) was added. After incubation, the patterns were washed by DI water and then dried by nitrogen. Two different biotinylated antibodies (Annexin A1 and EpCAM) were separately spotted on specific streptavidin-lipid patches by µCS. This was performed on an NLP 2000 system (NanoInk, Skokie, IL, USA) equipped with surface-patterning-tool (SPT) probes [50] (SPT-S-C10S, Bioforce Nanosciences, Ames, IA, USA). Before spotting, the SPTs were freshly plasma-cleaned by oxygen plasma (0.2 mbar, 100% O_2_, 100 W, 2 min, Atto plasma cleaner, Diener electronic, Ebhausen, Germany). Then, 20% of glycerol was added to the 1 µg µL^−1^ in PBS solutions of antibody, to avoid premature drying of ink on the SPT probes during spotting. After the loading of the SPT reservoir with ink, the probe was mounted, with a custom-made holder, into the NLP 2000, and the ink was spotted to the desired lipid patches in the arrays under manual control of the instrument. After spotting, samples were left for incubation, at room temperature, for 30 min, and then washed with DI water, to remove excess ink and unbound biotinylated antibody. For visualization, the lipid arrays were then incubated with 50 µL of fluorescently labeled secondary-antibody (goat anti-mouse IgG H&L (Alexa Fluor 488) and donkey anti-rabbit IgG H&L (Alexa Fluor 647)) of concentration 0.2 µg µL^−1^ in PBS. After 1 h of incubation, the sample was washed with DI water and inspected wet with fluorescent microscopy.

### 4.6. Optical Microscopy

All optical microscopy images (bright field and fluorescence) were obtained on an upright microscope setup (Eclipse 80i, Nikon, Düsseldorf, Germany), using the on-board software (NIS element, Nikon, Düsseldorf, Germany). Illumination for fluorescence microscopy was an Intensilight (Nikon, Düsseldorf, Germany), and filter cubes for FITC (excitation/emission wavelength: 475 nm/530 nm, color-coded green), Cy5 (604 nm/712 nm, color-coded purple) and TexasRed (559 nm/630 nm, color-coded red) were used.

## 5. Conclusions

We demonstrated the highly beneficial properties of block-type MPC copolymer substrates for use in L-DPN. These substrates allow for stable patterning of higher lipid membrane stacks in comparison to other hydrophilic substrates, and this is especially desirable in sensing applications for the potential of higher sensitivity. The stability and better visibility of the membranes’ patches on the block-type MPC copolymer substrate enables targeted functionalization of selected features in lipid arrays, here exemplified by a multiplexed delivery of antibodies via µCS. This allows for multiplexing regardless of availability of orthogonal binding tags for the antibodies. Combined with the antifouling/hemocompatible properties, the hydrophilic block-type MPC copolymer substrates show therefore great potential for lipid-based biomedical sensing and diagnostic platforms.

## Figures and Tables

**Table 1:** Roughness and contact angle of the different substrates.

Substrate	R_q_ (nm)	Contact Angle (°)
Glass	1.05 ± 0.08	27.5 ± 1.2 [26]
SiO_x_ (SEEC) [25]	0.22 ± 0.02	61.9 ± 3.3 [27]
Block-type MPC copolymer	0.43 ± 0.07	25.5 ± 1.8

**Table 2:** Average line width of lipid lines on the different substrates.

Substrate	Average Line Width (µm)
Glass	6.8 ± 0.7
SiO_x_ (SEEC)	5.5 ± 0.2
Block-type MPC copolymer	2.5 ± 0.1

## Supplementary Materials

The following are available online. Figure S1: Roughness data. Figure S2: Contact angle data. Figure S3: Average height of lipid structures. Figure S4: Fluorescence intensity during washing steps.
