# Supplementary material for: Enhanced Stability of Lipid Structures by Dip-Pen Nanolithography on Block-Type MPC Copolymer
Source: Molecules. 2020 Jun 15;25(12):2768. doi: 10.3390/molecules25122768 (PMC7356513; doi:10.3390/molecules25122768)
Supplement: Supplementary file 1 [file molecules-25-02768-s001.pdf]

Supplementary Materials for

Enhanced Stability of Lipid Structures by Dip-Pen Nanolithography on block-type MPC Copolymer

Hui-Yu Liu [1], Ravi Kumar [1], Madoka Takai [2] and Michael Hirtz [1,*]

[1] Institute of Nanotechnology (INT) & Karlsruhe Nano Micro Facility (KNMF), Karlsruhe Institute of Technology (KIT), Hermann-von-Helmholtz-Platz 1, 76344 Eggenstein-Leopoldshafen, Germany; hui-yu.liu@kit.edu (H.-Y.L.); ravi.kumar@kit.edu (R.K.)

[2] Department of Bioengineering, University of Tokyo, Japan; takai@bis.t.u-tokyo.ac.jp

* Correspondence: michael.hirtz@kit.edu; +49-721-6082-6373

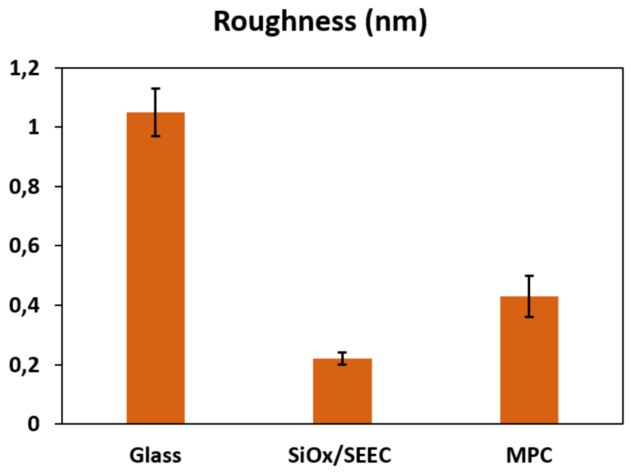

Figure S1. Comparison of roughness (R_q) as obtained from AFM measurements in air for the different substrate systems.

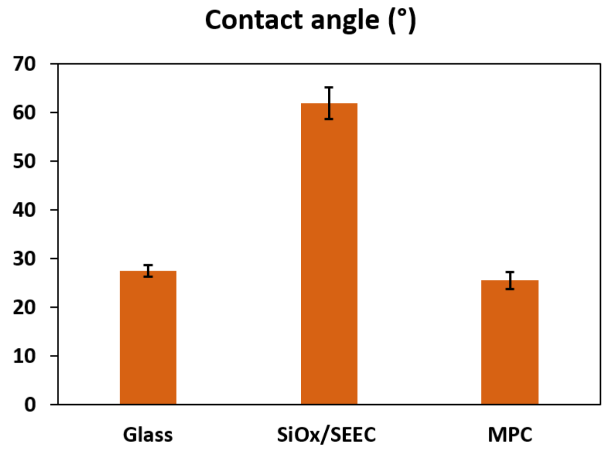

Figure S2. Comparison of water contact angle for the different substrate systems.

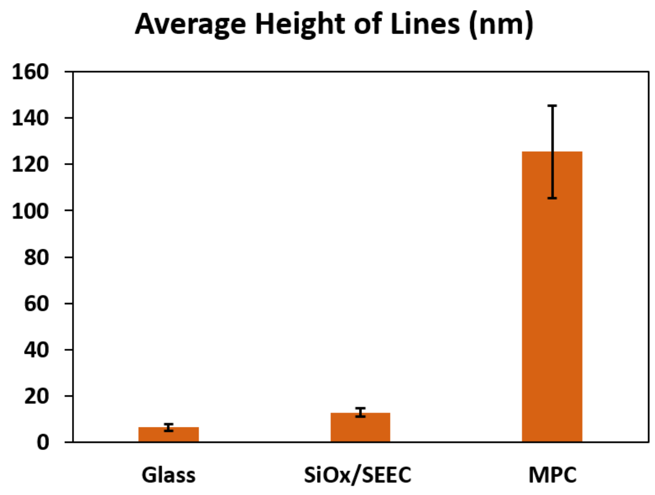

Figure S3. Comparison of the average height of the lipid structures on the different substrate systems as obtained by AFM measurements.

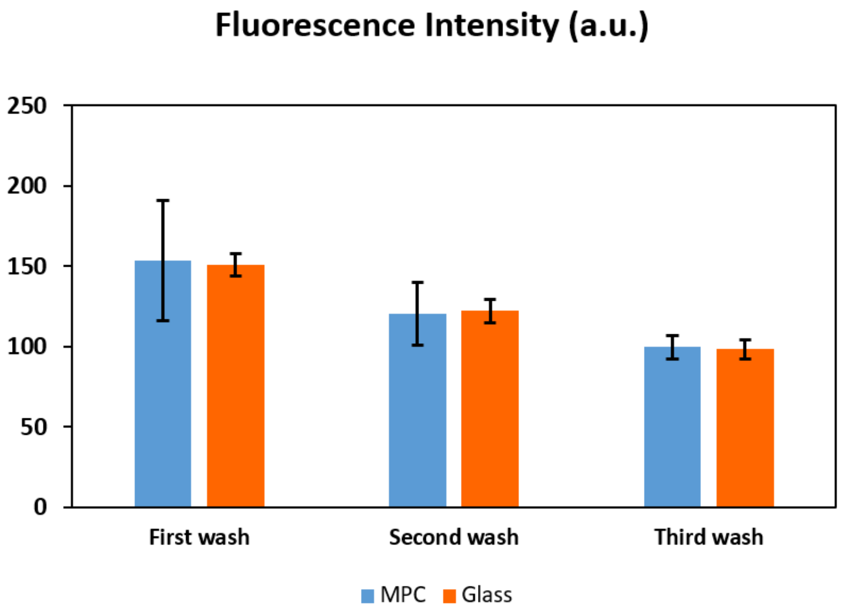

Figure S4. Fluorescence intensity on washing of structures for a fluorescently labelled lipid patch array on MPC copolymer or glass substrate.
